# Breast self-examination: Knowledge, practice and associated factors among 20 to 49 years aged women in Butwal sub-metropolitan, Rupandehi, Nepal

**DOI:** 10.1371/journal.pone.0286676

**Published:** 2023-06-02

**Authors:** Manisha B. K., Hari Prasad Kaphle

**Affiliations:** School of Health and Allied Sciences, Pokhara University, Pokhara, Nepal; Ambo University, ETHIOPIA

## Abstract

**Background:**

Breast cancer is the second most common cancer in the world and also among Nepalese women. Breast self-examination is an important, cheap, and easy method for early diagnosis of breast cancer which can be cured in the majority of cases if diagnosed in the early stages. In developing countries like Nepal where the awareness regarding breast cancer and breast self-examination is poor, breast cancers are diagnosed at late stages resulting in a poor prognosis of the disease. The study assessed knowledge, practice, and factors associated with breast self-examination.

**Methods:**

A cross-sectional survey was carried out among 262 women in the Butwal sub-metropolitan adopting multi-stage sampling. A pre-tested structured interview schedule and an observation checklist were used to collect the data. Data was entered in EPI-data and necessary univariate, bivariate, and multivariate analyses were done in SPSS.

**Results:**

The study found that more than half of the participants (55.3%) had poor knowledge of BSE. Only one-fourth (27.1%) of them were practicing BSE and among them, most of them (93.0%) had poor practice. The factors such as ethnicity from Brahmin/Chhetri [AOR = 2.099, 95% CI (1.106–3.981)], use of contraceptive devices [AOR = 9.487, 95% CI (2.166–41.558)], personal history of breast lump [AOR = 12.502, 95% CI (1.639–95.387)], family history of breast cancer [AOR = 5.729, 95% CI (1.337–97.512)], and knowledge of BSE [AOR = 4.407, 95% CI = 2.160–34.650)] were significant determinants of BSE practice among 20–49 years women.

**Conclusion:**

The study concluded that most of the women had poor knowledge and practice of breast self-examination. The study also indicated the influence of ethnicity, contraceptives, personal and family history of cancer/early warning signs, and knowledge for practicing breast self-examination. There is an immediate need to increase the knowledge and practice of breast self-examination to prevent and detect breast cancer in its early stage.

## Introduction

Breast cancer is a type of cancer that grows from breast cells and therefore the development starts from the inner lining of milk ducts (ductal carcinoma) or the lobules that offer milk (lobular carcinoma) [1]. It is the leading cause of cancer mortality among women in the world [2]. It’s the second commonest cancer among women in Nepal that is commonest in urban settings. It’s additionally common among young premenopausal women and plenty being diagnosed at a sophisticated stage [3]. Risk factors of breast cancer in Nepal are nulliparity or late age at first childbirth (>35 years of age), family history, smoking, excessive alcohol consumption, consumption of fat (BMI≥30Kg/M^2^), and exposure to radiation and hormone replacement therapy [4].

BC is the most common cancer in females, in both developing and developed countries, with an estimated 297,790 new cases and 43,170 deaths in the United States in 2023 [5]. There were an estimated 2.1 million newly diagnosed female breast cancer cases and 600 thousand breast cancer-related deaths occurred in 2018 [6]. In women, BC is the most commonly diagnosed cancer (11.7% of total cases) and the leading cause of cancer death (6.9%) in 110 countries among the 185 countries. It is the leading cause of global cancer incidence in 2020, with an estimated 2.3 million new cases, representing 11.7% of all cancer cases. It’s the fifth leading cause of cancer mortality in the world, with 685,000 deaths [7]. It accounts for one in four cancer cases and one in six cancer deaths, ranking first for incidence in the vast majority of countries (159 of 185 countries) and mortality in 110 countries [7]. In Nepal, according to the data from seven major cancer service hospitals in 2012, BC was the second most common cancer among women, after cancer of the cervix. According to GLOBOCAN 2012, an estimated 1,700 new BC cases were diagnosed in Nepal in 2012 with an age-standardized rate (ASR) of 13.7 new cases per 100,000 women, while 870 fatalities in women occurred with an ASR of 7.2 cases fatalities per 100,000 women [4]. GLOBOCAN 2018 estimates of cancer incidence and mortality, 2018 an estimated 2068 new BC cases were diagnosed in Nepal, with an ASR of fifteen cases per 100,000 women, whereas 1018 deaths occurred with an ASR of 7.6 cases per 100,000 women [8].

Early detection of breast cancer can reduce its morbidity and mortality. Mammography, clinical breast examination (CBE), and breast self-examination (BSE) are considered to be effective strategies for the early detection of breast cancer [9]. BSE is a patient-centered, cheap, and non-invasive method of screening for breast cancer, which improves the chances of early BC detection [2]. It’s a process whereby women examine their breasts frequently to detect any abnormal swelling or lumps to seek prompt medical attention. It’s one of the screening strategies used to detect breast-related issues like possible lumps, distortions, or swelling. BSE is usually recommended for each woman higher than the age of 20 years to be done for 20 minutes once monthly, between the seventh and tenth days of the menstrual cycle (2–3 days after the menses has gone), and goes an extended manner in detecting breast [10]. Early diagnosis has a positive impact on the prognosis and limits the development of complications and disability. It will increase life quality and survival [11]. In BSE, the patient observes and palpates their breasts and accessory anatomical structures to detect changes or abnormalities that will indicate the presence of cancer. Throughout the palpation of the breasts and adjacent structures (nipples, areolas, and axilla), lymph nodes and condensations also suggestive of neoplasias can be noticed [12]. Although BSE alone is not sufficient for early detection of breast carcinoma, it is still a very important screening tool for early detection of carcinoma in developing countries, because it is cheap, widely available, and doesn’t need complicated technical coaching, hospital visits, and specialized instrumentality [9].

BSE in conjointly with screening mammography is presently advocated by several organizations, and it’s also conjointly suggested for younger women beginning in their 20s who are nevertheless being screened by mammography [13]. Performing BSE is one method for a woman to understand how her breasts commonly feel so that she will notice any changes that do occur. Women who perform BSE monthly and properly are more likely to detect a lump within the early stage of its tumor. Evidence showed that comprehensive knowledge of BSE remains low in several developing countries [13].

This study aimed to assess knowledge, practice, and factors associated with breast self-examination among 20–49 years aged women in Butwal Sub-Metropolitan.

## Materials and methods

### Study design

The study design was cross-sectional.

### Study method

The study method was quantitative.

### Study setting

This study was conducted in the Butwal Sub-Metropolitan, Rupandehi district, Lumbini Province of Nepal. Since breast cancer is the most common cancer among women in urban settings [3], the Butwal sub-metropolitan is an urban area where the study on breast cancer and breast self-examination was inadequate.

### Study population

The study population was women aged 20 to 49 years who are currently residing in the Butwal Sub-Metropolitan of Rupandehi district as BSE is recommended to start from the age of early 20 and breast cancer is more common in young pre-menopausal women.

### Sample size

The prevalence of BSE practice in Butwal was 19.2% [14]. The required sample size was calculated with an assumption of 95% confidence interval (CI) & 5% maximum allowable error (d), the prevalence (p), and the population of 20–49 years women from the selected wards (N).

Using the sampling formula,

$$\text{n}=\frac{\frac{{\text{z}}^{2}\text{p}\left(\text{1}-\text{p}\right)}{{\text{d}}^{2}}}{1+\left[\frac{{\text{z}}^{2}\text{p}\left(1-\text{p}\right)}{{\text{d}}^{2}\text{N}}\right]}$$

Here, n = required sample size, value of z at 95% confidence level = 1.96, d = 0.05, p = 19.2% = 0.192, q = 1-p = 1.0–0.192 = 0.808, and N = 22,332. Putting these values in the above formula we got n = 235.87 (n≈ 236) and adding a 10% non-response rate, the final sample size for the study was 262.

### Sampling procedure

Multi-stage sampling technique was used for this study. In the first stage, among 19 wards of Butwal Sub-Metropolitan, 6 wards (33%) were selected randomly by simple random sampling. In the second stage, the required number of participants was identified based on proportionate sampling (Table 1). In the third stage, the participants to be included in the study were selected using systematic random sampling. The first household was selected by choosing a random number from 0 to 9. Another random number from 0 to 9 was selected for determining the interval. In a selected household, if there were more than one eligible woman then the participant with the highest age was chosen.

**Table 1:** Sampling frame/technique.

Ward No	Estimated population of 20–49 years women (Household statistics, 2076)	Required sample size (1.17%)
5	718	8
6	1,398	17
8	2,652	31
11	12,130	142
13	3,735	44
17	1,699	20
Total	22,332	262

### Selection criteria

#### Inclusion criteria

- Women of age between 20 to 49 years who were residing in the Butwal sub-metropolitan of Rupandehi district, Lumbini Province Nepal, and provided consent to participate in the study.

#### Exclusion criteria

- Those women of age between 20 to 49 years who were unable to respond due to severe physical and mental problems.
- Those women had a history of breast cancer or mastectomy.

### Ethical considerations

Ethical approval was obtained from the Institutional Review Committee, Pokhara University with reference number 11/078/079. Permission was also taken from the Butwal Sub-Metropolitan. Participants were fully informed about the objectives and purpose of the study before the data collection. Both verbal and written consent was taken. Confidentiality and privacy were maintained properly. The right to refuse to take part in the study was highly respected.

### Research tools and their development

A pre-tested structured interview schedule and an observational checklist were used for data collection. It was developed after an extensive literature review and consultation with experts.

The interview schedule included socio-demographic factors, gynecological and obstetric factors, knowledge, and self-reported practice of BSE.

Socio-demographic factors were age, marital status, religion, ethnicity, occupation, education, and household average monthly income. Gynecological and obstetric factors were age at marriage, parity, breastfeeding, contraceptive use, personal history of breast lump, family history of breast cancer, and mastectomy in the family.

Knowledge of BSE was assessed by self-constructed 10 multiple choice questions with three wrong and one right option on different dimensions of knowledge of BSE (see respective table in results for questions). Responses of the participants were categorized into 1 and 0 for right and wrong answers respectively and the mean knowledge score was calculated. Participants who performed below the mean score were classified as having poor knowledge and those who had mean or above mean were classified as having good knowledge of BSE.

Self-reported practice related to BSE was measured by self-constructed 14 different questions included in the interview schedule (see respective table in results for questions).

To observe the practice of BSE, an observation checklist was adopted from an Iranian study [15]. A female dummy was taken during the data collection to observe the steps of breast self-examination among those who practice. Twenty-two steps (see respective table in results for questions) were observed considering the dummy privately in a quiet room during the observation for breast self-examination practice. Participants were asked to show the steps on a dummy on how they performed breast self-examination. The scores ranged from 0 to 22 and each step was ticked if found to show in a dummy. The results were determined as good (15–22), medium (9–14), and poor (0–8).

### Pretesting, validity, and reliability

Pre-testing was done in a similar setting on 10% (26) of the sample size excluding the study area and changes were made accordingly. The interview schedule and observation checklist were designed based on the reference to various research papers. The data collection tool was made in simple, clear, and Nepali language. The research guide and experts were consulted for appropriate suggestions on design and tools. The researcher herself was involved in data collection and analysis. The validity and reliability were confirmed by the supervisor, experts, and pre-testing.

### Data collection procedure

Data were collected through face-to-face interviews and observation methods between April–May 2022. A structured questionnaire was used for the interview and an observational checklist for observing BSE practice. A female dummy was taken during the data collection to observe the steps of breast self-examination among those who practice. Data was gathered in the prescribed format. The interview was conducted after taking both verbal and written consent. The objective and purpose of the research were clearly described before taking consent. Confidentiality was also maintained. The Nepali language was used to collect the data and data collection was carried out following precautions against COVID-19.

### Data analysis

Epi-Data software was used for data entry and analysis was performed with the help of the Statistical Package for Social Science (SPSS). Descriptive statistics (i.e. frequency, percentage, mean and standard deviation) were applied to describe the study population. Pearson’s Chi-square test was used to find out the association between dependent and independent variables i.e. association between the practice of BSE and socio-demographic, gynecological & obstetric, and knowledge-related factors. Bivariate logistic regression analysis was used among the associated factors. Multivariate logistic regression analysis was used to assess the predictors for the practice of BSE.

## Results

### Univariate analysis

#### Socio-demographic information

The majority of the participants were of the age group 40–49 years (Mean & SD: 37.28±7.689), belonged to Brahmin/Chhetri ethnicity (37.8%) and Hindu religion (82.5%), and had a secondary level of education (27.9%). Similarly, the majority of participants were from nuclear families (59.5%), housewives (43.5%), and married (81.7%). Moreover, the monthly household income of about three-quarters (77.5%) was less than fifty thousand (Mean & SD: 41,431±18,900) (Table 2).

**Table 2:** Sociodemographic characteristics of the participants (n = 262).

Variables	Frequency (n)	Percentage (%)
**Age (in years)**		
20–29	50	19.1
30–39	98	37.4
40–49	114	43.5
Mean±SD: 37.28±7.689 (Min = 21, Max = 49)		
**Ethnicity**		
Brahmin/Chhetri	99	37.8
Janajati	65	24.8
Madhesi	29	11.1
Dalit	43	16.4
Others (Musalman)	26	9.9
**Religion**		
Hindu	216	82.5
Christian	15	5.7
Muslim	26	9.9
Buddhist	5	1.9
**Education**		
Illiterate	60	22.8
Basic level	72	27.5
Secondary level	73	27.9
Higher education	57	21.8
**Family type**		
Nuclear	156	59.5
Joint	106	40.5
**Occupation**		
Housewife	114	43.5
Agriculture	28	10.7
Services	64	24.4
Business	28	10.7
Daily wages/Labor	17	6.5
Others	11	4.2
**Marital status**		
Unmarried	26	9.9
Married	214	81.7
Divorced	5	1.9
Widow	17	6.5
**Household average monthly income**		
<50,000	203	77.5
≥50,000	59	22.5
Mean±SD = 41,431±18,900 (Min = 5,000 Max = 1,00,000)		

#### Gynecological and obstetric information

More than half of the participants were married (59.3%), had ≤2 children (50.6%), and didn’t use any contraceptives (58.1%). Moreover, 13.4% had a history of breast cancer, and 6.5% and 4.2% had a family history of breast cancer and mastectomy respectively. Almost half (50.0%) had heard about BSE and the main source of information about BSE was the internet (46.6%) followed by healthcare providers (42.7%) respectively (Table 3).

**Table 3:** Gynecological and obstetric information.

Variables	Frequency (n)	Percentage (%)
**Age at marriage (n = 236)**		
<20 years	140	59.3
≥20 years	96	40.7
Mean±SD: 19.46±3.075 (Min = 13, Max = 40)		
**Number of children (n = 225)**		
≤2	114	50.6
>2	111	49.4
Mean±SD: 2.59±1.006 (Min = 1, Max = 6)		
**Duration of breastfeeding to the last child (n = 225)**		
Up to 2 years	67	29.8
>2 years	158	70.2
Mean±SD: 3.01±0.935 (Min = 1, Max = 5)		
**Use of any contraceptive devices (n = 236)**		
Yes	99	41.9
No	137	58.1
**Mainly used method (n = 99)**		
Condom	21	21.2
Depo-Provera	14	14.2
IUCD	24	24.2
Norplant	6	6.1
OCP	34	34.3
**Personal history of a breast lump (n = 262)**		
Yes	35	13.4
No	227	86.6
**Family history of breast cancer (n = 262)**		
Yes	17	6.5
No	245	93.5
**Family history of mastectomy for breast cancer (n = 262)**		
Yes	11	4.2
No	251	95.8
**Heard about BSE (n = 262)**		
Yes	131	50.0
No	131	50.0
**The main source of information about BSE (n = 131)**		
Health care providers	56	42.7
Internet	61	46.6
Neighbors	9	6.9
TV	5	3.8

#### Knowledge of BSE

Out of the 262 participants, more than half of them (55.3%) had poor knowledge of BSE (Table 4).

**Table 4:** Knowledge-related information (n = 262).

S.N.	Knowledge of different dimensions of BSE	Frequency (n)	Percentage (%)
1.	Why do women have to perform BSE? (to detect any abnormal changes in the breast earlier)	141	53.8
2.	At what age do women have to start BSE? (from 20 years)	66	25.2
3.	How frequently do women have to perform BSE? (monthly)	183	69.8
4.	On which days of the menstrual cycle woman have to perform BSE if she has a regular cycle? (7–10 days of menstruation)	81	30.9
5.	On which days of the menstrual cycle do women have to perform BSE if she has an irregular cycle? (on the same day in each month)	203	77.5
6.	What to see mainly in the breast while performing BSE in front of a mirror? (swelling, dimpling of the skin, or changes in the nipples)	151	57.6
7.	How to palpate the breasts while performing BSE? (use the right hand for the left and the left hand for the right breast)	148	56.5
8.	What is the correct direction for palpation? (circular)	53	20.2
9.	What to feel mainly while performing BSE? (lumps, hard knots, or thickening)	165	63.0
10.	How to look for any discharge? (by squeezing the nipple of the breast)	134	51.1
**Level of knowledge on BSE**			
1.	Good	117	44.7
2.	Poor	145	55.3
**Mean±SD: 5.170±2.27 (Min = 0, Max = 10)**			

#### The self-reported practice of BSE

Based on the self-reported practice among 262 participants, only 27.1% of participants were practicing BSE in the last twelve months. However, among 27.1% who were practicing, only 59.3% started at the correct age, 36.6% were practicing BSE at the right frequency, and 53.6% were performing at right time. However, nearly all (94.4%) of them reported they were examining both breasts (Table 5).

**Table 5:** Practice-related information on BSE.

Variables	Frequency (n)	Percentage (%)
**Have you been practicing BSE in the last year? (n = 262)**		
Yes †	71	27.1
No	191	72.9
**At what age have you started BSE? (n = 71)**		
20–29 years †	42	59.3
30–39 years	24	33.7
40–49 years	5	7.0
**How often do you practice BSE? (n = 71)**		
Daily	4	5.6
Weekly	1	1.4
Monthly †	26	36.6
Half-yearly	40	56.4
**When do you practice BSE? (n = 71)**		
During menstrual flow	26	36.6
A week after period †	38	53.6
Before menstrual flow	5	7.0
During breastfeeding	2	2.8
**How do you practice BSE? (n = 71)**		
Palpate with one finger	0	0.0
Palpate with palm	2	2.8
Palpate with palm and 3 fingers †	59	83.1
Anyhow	10	14.1
**When examining your breast, what type of pattern do you use? (n = 71)**		
No pattern	17	23.9
Vertical strips	4	5.6
Circular †	50	70.5
Wedge	0	0.0
**Which hand do you use to examine your breast? (n = 71)**		
Use the right hand to examine the right breast and the left hand to examine the left breast	7	9.9
Use the right hand for both breasts	10	14.1
Use left hand for both breasts	0	0.0
Use the right hand to examine the left breast and the left hand to examine the right breast †	54	76.0
**When examining the breast, which area do you examine? (n = 71)**		
Axilla and breast	29	40.8
Breast only	41	57.8
The entire area that extends from the breast, up the breast bone area and collar area †	1	1.4
Breast and up the breast bone area	0	0.0
**When examining the breast, do you look at the mirror? (n = 71)**		
Never look in the mirror	35	49.3
Sometimes	20	28.2
During pain	13	18.3
Always look in the mirror †	3	4.2
**When looking in the mirror, in what position do you perform BSE? (n = 36)**		
Hands keeping at the hip	7	19.4
Hands keeping at head	18	50.0
Hands keeping on the side, hip, and head †	0	0.0
Hands keeping on side	11	30.6
**Do you lie on your side when examining the outside area of your breasts? (n = 71)**		
Never lie on the side	62	87.3
Sometimes lie on the side	8	11.3
During pain	1	1.4
Always lie on the side †	0	0.0
**Do you lie on your back to examine your breasts? (n = 71)**		
Never lie on the back	30	42.3
Sometimes lie on the back	28	39.4
During pain	12	16.9
Always lie on the back †	1	1.4
**When examining your breasts, do you move your fingers in a small circle? (n = 71)**		
Never use small circles	41	57.7
Sometimes	11	15.5
During pain	15	21.2
Always use small circles †	4	5.6
**When examining your breasts, do you examine both breasts? (n = 71)**		
Never examine both breasts	0	0.0
Sometimes examine both breasts	0	0.0
During pain	4	5.6
Always examine both breasts †	67	94.4

^†^indicates the desired practices.

#### The practice of BSE on observation

However, the practice of BSE on observation on a female dummy revealed that nearly all of them have a poor practice of BSE (93.0%) (Table 6).

**Table 6:** Observation checklist (n = 71).

S.N.	Variables	Yes	No
		n (%)	n (%)
1.	For BSE, she uses to observe them in front of the mirror	36 (50.7)	35 (49.3)
2.	For BSE, she assesses both breasts by observation in terms of shape and similarity	11 (15.5)	60 (84.5)
3.	For BSE, she assesses both breasts by observation in terms of size and appearance	11 (15.5)	60 (84.5)
4.	She considers breast dimples and nipple sore	6 (8.5)	65 (91.5)
5.	To observe, she puts the arms on the sides of the body	34 (47.9)	37 (52.1)
6.	To observe, she holds the dummy’s arms up	33 (46.5)	38 (53.5)
7.	To observe, she holds the dummy’s arms behind the head	19 (26.8)	52 (73.2)
8.	To observe, she puts the dummy’s arms behind the body	20 (28.2)	51 (71.8)
9.	She does all of the four above cases	5 (7.0)	66 (93.0)
10.	For self-examination, she touches the breasts	71 (100)	0 (0.0)
11.	Breast touch is done by supine position	2 (2.8)	69 (97.2)
12.	In a supine position, a pillow is placed under the breasts	0 (0.0)	71 (100)
13.	In a supine position, the hand of the dummy is put under the head	0 (0.0)	71 (100)
14.	To touch, the tip of fingers are used	15 (21.1)	56 (78.9)
15.	Breast touch is done using rotary movements of the fingers	5 (7.0)	66 (93.0)
16.	Breast touch is done using longitudinal movements of the fingers	0 (0.0)	71 (100)
17.	Breast touch is done using radial movements of the fingers	0 (0.0)	71 (100)
18.	All of the above movements are done	0 (0.0)	71 (100)
19.	She presses the nipples for any type of tumor and blood discharge	4 (5.6)	67 (94.4)
20.	Touching the upper outer quadrant of the breast (armpit side) is given very importance	7 (9.9)	64 (90.1)
21.	Touching the lymph glands in the armpit is given importance	0 (0.0)	71 (100)
22.	Touching the lymph nodes in supraclavicular is given importance	0 (0.0)	71 (100)
	**Practice level**		
	Good	0	0.0
	Medium	5	7.0
	Poor	66	93.0

### Bivariate analysis

Among the socio-demographic variables, the result showed that age, education, family type, occupation, household average monthly income, and marital status were significantly associated with the practice of BSE in Parson’s chi squire test (p<0.05) (Table 7).

**Table 7:** Association of socio-demographic variables with the practice of BSE.

Variables	Practice of Breast self-examination (BSE)		Chi-square value	df	p-value
	Yes	No			
	71 (27.1%)	191 (72.9%)			
**Age (in years)**					
20–29	19 (38.0)	31 (62.0)	28.191	2	<0.001*
30–39	40 (40.8)	58 (59.2)			
40–49	12 (10.5)	102 (89.5)			
**Ethnicity**					
Brahmin/Chhetri	35 (35.4)	64 (64.6)	5.488	1	0.019*
Others	36 (22.1)	127 (77.9)			
**Religion**					
Hindu	58 (26.9)	158 (73.1)	0.038	1	0.845
Others	13 (28.3)	33 (71.7)			
**Education**					
Illiterate	3 (5.0)	57 (95.0)	35.598	2	<0.001*
Basic level	12 (16.7)	60 (83.3)			
Secondary & higher education	56 (43.1)	74 (56.9)			
**Family type**					
Nuclear family	51 (32.7)	105 (67.3)	6.106	1	0.013*
Joint family	20 (18.9)	86 (81.1)			
**Occupation**					
Housewife	18 (15.8)	96 (84.2)	16.682	2	<0.001*
Agriculture	6 (21.4)	22 (78.6)			
Business & Others	47 (39.2)	73 (60.8)			
**Household average income**					
<50,000	42 (20.7)	161 (79.3)	18.746	1	<0.001*
≥50,000	29 (49.2)	30 (50.8)			
**Marital status**					
Unmarried	13 (50.0)	13 (50.0)	7.663	1	0.006*
Married	58 (24.6)	178 (75.4)			

*Statistically significant at the level of p-value<0.05

Among the gynecological and obstetric variables, age at marriage, number of children, use of contraceptive devices, personal history of breast lump, family history of breast cancer, and family history of mastectomy were significantly associated with the practice of BSE in Parson’s chi squire test (p<0.05) (Table 8).

**Table 8:** Association of gynecological and obstetrical variables with the practice of BSE.

Variables	Practice of Breast self-examination (BSE)		Chi-square value	df	p-value
	Yes	No			
	71 (27.1%)	191 (72.9%)			
**Age at marriage (n = 236)**					
< 20 years	26 (15.9)	138 (84.1)	22.064	1	<0.001*
≥ 20 years	32 (44.4)	40 (55.6)			
**No. of children (n = 225)**					
≤ 2 years	44 (38.6)	70 (61.4)	23.226	1	<0.001*
> 2years	12 (10.8)	99 (89.2)			
**Duration of breastfeeding to the last child (n = 225)**					
Up to 2 years	12 (17.9)	55 (82.1)	2.485	1	0.115
> 2 years	44 (27.8)	114 (72.2)			
**Use of contraceptive devices (n = 236)**					
Yes	36 (36.4)	63 (63.6)	12.783	1	<0.001*
No	22 (16.1)	115 (83.9)			
**Personal history of breast lump (n = 262)**					
Yes	18 (51.4)	17 (48.6)	12.104	1	0.001*
No	53 (23.3)	174 (76.7)			
**Family history of breast cancer (n = 262)**					
Yes	12 (70.6)	5 (29.4)	17.404	1	<0.001*
No	59 (24.1)	186 (75.9)			
**Family history of mastectomy (n = 262)**					
Yes	8 (72.7)	3 (27.3)	12.100	1	0.001*
No	63 (25.1)	188 (74.9)			

*Statistically significant at the level of p-value<0.05

Moreover, the result showed that the level of knowledge was significantly associated with the practice of BSE (p<0.05) (Table 9).

**Table 9:** Association of level of knowledge of BSE with the practice of BSE.

Variables	Practice of Breast self-examination (BSE)		Chi-square value	df	p-value
	Yes	No			
	71 (27.1%)	191 (72.9%)			
**Level of knowledge**					
Good	52 (44.4)	65 (55.6)	32.195	1	**<0.001** *
Poor	19 (13.1)	126 (86.9)			

*Statistically significant at the level of p-value<0.05

The strength of the association of statistically significant factors with the practice of BSE in bivariate analysis logistic regression analysis is presented below (Table 10).

**Table 10:** Factors associated with BSE in bivariate logistic regression analysis.

Variables	UOR	95% CI	p-value
**Socio-demographic variables**			
**Age (in years)**			
20–29	5.826	(2.850–12.650)	<0.001*
30–39	5.210	(2.279–11.911)	<0.001*
40–49	Reference		
**Ethnicity**			
Brahmin/Chhetri	1.929	(1.109–3.357)	0.020*
Others	Reference		
**Education**			
Illiterate	Reference		
Basic level	3.800	(1.019–14.169)	<0.001*
Secondary & Higher education	14.378	(4.280–48.303)	<0.001*
**Family type**			
Nuclear	2.089	(1.157–3.770)	0.014*
Joint	Reference		
**Occupation**			
Housewife	Reference		
Agriculture	2.361	(0.891–6.254)	0.084
Business & Others	3.434	(1.842–6.401)	<0.001*
**Household average monthly income (NRs)**			
<50000	Reference		
≥50000	3.706	(2.007–6.841)	<0.001*
**Marital status**			
Unmarried	0.326	(0.143–0.743)	0.008*
Married	Reference		
**Gynecological and obstetrical variables**			
**Age at marriage (years)**			
<20	Reference		
≥20	4.246	(2.271–7.939)	<0.001*
**Number of children**			
≤2	5.186	(2.555–10.525)	<0.001*
>2	Reference		
**Use of contraceptive devices**			
Yes	2.987	(1.618–5.514)	<0.001*
No	Reference		
**Personal history of breast lump**			
Yes	3.476	(1.674–7.218)	0.001*
No	Reference		
**Family history of breast cancer**			
Yes	7.566	(2.560–22.360)	<0.001*
No	Reference		
**Family history of mastectomy**			
Yes	7.958	(2.048–30.919)	0.003*
No	Reference		
**Level of Knowledge**			
**Knowledge of BSE**			
Good knowledge	5.305	(2.898–9.712)	<0.001*
Poor knowledge	Reference		

*Statistically significant at the level of p-value<0.05

### Multivariate analysis

On multivariate analysis after adjustment of covariates, participants from Brahmin/Chhetri ethnicity were two times more likely [AOR = 2.099, 95% CI (11.106–3.981)] to practice BSE as compared to others. Participants who use contraceptive devices were nine times more likely [AOR = 9.487, 95% CI (2.166–41.558)] to practice BSE as compared to those who didn’t use it. Participants who had a personal history of breast lumps were twelve times more likely [AOR = 12.502, 95% CI (1.639–95.387)] to practice BSE as compared to those who didn’t. Participants who had a family history of breast cancer were five and half times more likely [AOR = 5.729, 95% CI (1.337–97.512)] to practice BSE as compared to those who didn’t have. Furthermore, participants who had good knowledge were about four and half times more likely [AOR = 4.407, 95% CI = 2.160–34.650)] to practice BSE as compared to those who had poor knowledge (Table 11).

**Table 11:** Factors associated with the practice of BSE in multivariate logistic regression analysis.

Variables	AOR	95% CI	p-value
**Socio-demographic variables**			
**Age (in years)**			
20–29	1.706	(0.165–17.586)	0.654
30–39	0.748	(0.152–3.674)	0.721
40–49	Reference		
**Ethnicity**			
Brahmin/Chhetri	2.099	(1.106–3.981)	0.023*
Others	Reference		
**Education**			
Illiterate	Reference		
Basic level	0.482	(0.011–21.905)	0.708
Secondary & Higher education	4.099	(0.560–29.993)	0.165
**Family type**			
Nuclear	1.813	(0.440–7.467)	0.410
Joint	Reference		
**Occupation**			
Housewife	Reference		
Agriculture	0.066	(0.003–1.574)	0.093
Business and others	0.353	(0.068–1.823)	0.214
**Household average monthly income**			
<50000	Reference		
≥50000	1.309	(0.264–6.486)	0.741
**Marital status**			
Unmarried	0.939	(0.340–2.588)	0.903
Married	Reference		
**Gynecological and obstetrical variables**			
**Age at marriage**			
<20	Reference		
≥20	2.349	(0.460–12.003)	0.305
**Number of children**			
≤2	2.844	(0.485–14.619)	0.264
>2	Reference		
**Use of contraceptive devices**			
Yes	9.487	(2.166–41.558)	0.003*
No	Reference		
**Personal history of breast lump**			
Yes	12.502	(1.639–95.387)	0.015*
No	Reference		
**Family history of breast cancer**			
Yes	5.729	(1.337–97.512)	0.027*
No	Reference		
**Family history of mastectomy**			
Yes	171.892	(0.437–676.104)	0.091
No	Reference		
**Level of Knowledge**			
**Level of knowledge**			
Good	4.407	(2.160–34.650)	0.049*
Poor	Reference		

*Statistically significant at a level of p-value<0.05

## Discussion

The finding of this study indicates that most of the reproductive age group women had poor knowledge of BSE. Only 44.7% had good knowledge of BSE. A similar study conducted at Rapti Sonari Rural Municipality, Banke District showed about the same proportion of women had good knowledge (44.3%) [16]. Similarly, other studies conducted in Ghana (43.3%) [13], Southwest Ethiopia (30.0%) [10], and Southwest Cameroon (25.4%) [17] also reported less proportion of having good knowledge of BSE. The reason for the difference in knowledge in different studies from different countries might be due to the difference in the study setting and sociocultural factors across the world.

Moreover, the current study also showed that 27.1% of women were practicing BSE. A similar study in the Banke district of Nepal showed 19.6% of women were practicing BSE [16]. Similarly, studies conducted in the South district of Ghana (27.5%) [13], Tabriz, Iran showed (18.8%) [15], and Northwest, Ethiopia (32.5%) [18] showed a lower proportion were practicing BSE. However urban women of Shah Alam, Malaysia higher (55.0%) % of women were practicing BSE [19]. Studies from various countries showed somehow differences in the practice of BSE. The reason for this might be due to the difference in knowledge of study participants and the difference between study areas.

In observation, none of the women had good practice of BSE in this study. A similar study conducted among women in Tabriz, Iran showed performance of practice was very poor among 22.7%, poor among 46.7%, medium among 21.3%, good among 6.7%, and very good among 2.7% (17). It indicates that even though some women have knowledge of BSE and practice it, however, they aren’t practicing following the appropriate technique.

After adjustment of covariates in the Multivariate logistic regression analysis ethnicity (Brahmin/Chhetri) [AOR = 2.099, 95% CI (1.106–3.981)], use of contraceptive devices [AOR = 9.487, 95% CI (2.166–41.558)], personal history of breast lump [AOR = 12.502, 95% CI (1.639–95.387)], family history of breast cancer [AOR = 5.729, 95% CI (1.337–97.512)], and knowledge on BSE [AOR = 4.407, 95% CI = 2.160–34.650)] were significant predictors of the practice of BSE.

The finding of the current study showed ethnicity as a significant determinant for practicing BSE which in contrast with the study conducted among women of reproductive age of Rapti Sonari rural municipality, Banke district, Nepal that there was no significant difference [16]. This contrasting finding might be due to the differences in knowledge in different ethnic groups living in the two study settings.

Similarly, the current study showed the use of contraceptive devices as a significant determinant for practicing BSE which showed a contrast finding with the study conducted among women who attended primary health care, in Kuwait that there was no significant difference in BSE practice regarding the use of contraceptive devices [20]. This contrasting finding might be due to the reason that those who use contraceptive devices get health education regarding reproductive health and disease from the health institution.

Likewise, the current study revealed that personal history of breast lumps is a significant determinant for practicing breast self-examination (AOR = 12.505, 95% CI = 1.639–95.387). The finding of this study is supported by the study conducted in Ethiopia in 2018, where women with a personal history of breast problems were 3.27 times more likely to practice BSE [21]. This finding is also supported by another study conducted in Iran in 2018 [22]. This might be the reason that one with a breast lump or disease gets information and advice from the health institution and are conscious about their health disease.

Furthermore, the finding of this study revealed that a family history of breast cancer was also a significant determinant for practicing BSE (AOR = 5.729, 95% CI = 1.337–97.512). The finding of this study is supported by different studies [18, 23, 24]. This could be due to the reason of getting information and advice from health care providers and being aware as they might be at risk for breast cancer, so it can be detected earlier with breast self-examination.

In addition, this study showed women’s knowledge as a significant determinant for practicing BSE (AOR = 4.407, 95% CI = 2.160–34.650). This finding is supported by the various studies (AOR = 5.74) [18], (AOR = 4.32) [10], and (AOR = 12.02) [23]. This indicates that knowledgeable women motivate themselves to practice breast self-examination.

## Conclusions

The study concludes that most of the women had poor knowledge. Also, very few women were practicing breast self-examination. Among those who were practicing breast self-examination, almost all of them demonstrated poor practice. Moreover, the study also concludes that ethnicity, use of contraceptives, personal history of breast lump, family history of breast cancer, and level of knowledge have a significant influence on practicing breast self-examination. There is an immediate need to increase the knowledge and practice of breast self-examination to prevent and detect breast cancer in its early stage.

## Limitations

The study was carried out in selected wards of the Butwal sub-metropolitan of the Rupandehi district of Nepal and therefore, it might not be representative of the entire country. Likewise, the proportion of women practicing BSE in this study was assessed by a self-reported response regarding whether they have been practicing BSE in the last year or not. Other aspects of the practice such as frequency, timing, process, etc. of BSE weren’t considered while determining the factors associated with the practice of BSE.

## Supporting information

S1 Dataset (SAV)
