# Supplementary material for: Breast self-examination: Knowledge, practice and associated factors among 20 to 49 years aged women in Butwal sub-metropolitan, Rupandehi, Nepal
Source: PLoS One. 2023 Jun 2;18(6):e0286676. doi: 10.1371/journal.pone.0286676 (PMC10237491; doi:10.1371/journal.pone.0286676)
Supplement: Attachment — Submitted filename: Response to Reviewers.docx [file pone.0286676.s002.docx]

**Response to Reviewer**

Manuscript#: PONE-D-22-25228
Title of Manuscript: Breast Self-Examination: Knowledge, Practice and Associated factors among 20 to 49 years aged Women in Butwal Sub-Metropolitan, Rupandehi, Nepal.

Note: page numbers/line numbers describe here are page/line numbers of revised manuscript without track change. The page number/line number may be different in revised manuscript with track change.

| Reviewers comments | Authors Responses |
| --- | --- |
| **Reviewer #1** This manuscript is interesting because it deals with the most common disease in low-income countries. Because BSE is free and simple to perform, it has the potential to save women's lives. However, there are a few comments on this paper: |  |
| 1- Based on the study design, inclusion criteria are insufficient; what about those who cannot read or write, if any? | Thank you for your comment. Those who were literate and those who cannot read and write are all included in the study since the required information was collected through a face-to-face interview conducted by ourselves. However, inclusion and exclusion criteria are corrected to show clarity to the readers. Please see pages 6 and 7 line numbers 123-131 in the revised manuscript. |
| 2- The original questionnaire used in this study is not mentioned or acknowledged. | Thank you for your valuable comment. Now we have acknowledged it. Please see page no 8 line number 155 in the revised manuscript. |
| **Reviwer#2**: I would like to thank the author for selecting an important research topic for publication. However, I would like to share with you some comments to be reviewed and corrected accordingly. |  |
| 1- Introduction, page 2, line 38: Late menarche is associated with a decreased risk of developing breast cancer in later life but not included as risk factors for breast cancer. | Thank you for your valuable comment. Now it has been removed from the revised manuscript. Please see page no 2 line numbers 42-45 in the revised manuscript. |
| 2- Page 1. Introduction, line 40: Breastfeeding is lowering the risk of developing breast cancer, particularly if you have your children when you are younger. The longer you breastfeed the more the risk is reduced. It is not as you mentioned in your sentence. Please revise this information. | Thank you for your valuable comment. Evidence showed that breastfeeding lowers the risk of breast cancer. However, the contrasting result presented by the literature we have cited. We have removed this contrast finding in the revised manuscript. Please see page no 2 line numbers 42-45 in the revised manuscript. |
| 3- Page 1, line 41: "an estimated BC of 1.67 million new cases diagnosed in 2012" This is old data. Please make citation of recent data for example for the year 2022. | We acknowledge your valuable comments. Now it is replaced with new data. Please see page no 3 line numbers 46-47 in the revised manuscript. |
| 4- Page 3, line 82: "But, this examination is crucial for Ghanaian women as a result of black women are found in reality the bigger burden of carcinoma mortality compared to alternative races (13)." I didn't think this sentence is relevant in the text. | We accept your comment. It is removed in the revised manuscript. |
| 5- Page 3, line 86: "Evidence showed that comprehensive knowledge of BSE remains low in several developing countries." this part needs reference. | Now we have added the citation and reference for the sentences. Please see page no 4 line numbers 86,87 in the revised manuscript. |
| 6- Page 5, line to 17: if it is possible to narrate all these information in a continuous sentences than to be in separate lines. | Thank you for your valuable suggestion. Now it is written in a continuous sentence. Please see pages no 5 & 6 line numbers 105-122 in the revised manuscript. |
| 7- Page 8, line 162, Data collection procedure: Observational checklist for observing BSE practice. Description of the main contents of the checklist should be added | We appreciate your valuable suggestion. Now it is included in the revised manuscript.  Please see page no 8 line numbers 155-162 in the revised manuscript. |
| 8- Page 9, line 172. Data analysis: "Association between dependent and independent variable such as: "should be mentioned. | It is mentioned in the revised manuscript. Please see page no 9 line numbers 182-185 in the revised manuscript. |
| 9- Page 12, result, line 201 and further. The narration of the result on table 4 needs to be summarized in form that to highlight the correlated information in the analysis. | We appreciate your valuable feedback. We have revised it based on your feedback. Please see page no 12 line numbers 205 and 206 in the revised manuscript. And also see table 4 in the revised manuscript. |
| 10-Page 13, result, line 225 and further. The narration of the result on table 5 needs to be summarized in form that to highlight the correlated information in the analysis. | We appreciate your valuable feedback. We have revised it based on your feedback. Please see page no 15 line numbers 209-213 in the revised manuscript. And also see table 5 in the revised manuscript. |
| 11- Page 16, result, line 242 and further. The narration of the result on table 6 needs to be summarized in form that to highlight the correlated information in the analysis. | We appreciate your valuable feedback. We have revised it based on your feedback. Please see page no 19 line numbers 217 and 218 in the revised manuscript. And also see table 6 in the revised manuscript. |
| 12- Page 23, line 330: this part should not be in this section, probably in the previous section when describing the Unadjusted OR. "An adjusted odds ratio was obtained by entering all the independent variables under different categories significantly associated with the chi-square test using the enter method in binary logistic regression analysis. | Thank you for your valuable feedback. Now this portion is removed from this section. Please see it in the revised manuscript (without track change) page no 28 line no 241. |
| 13- Discussion, page 25, line 250: the second paragraph of the discussion part needs to be re-phrased in better summarized way than it is... | Dear Reviewer, based on your valuable comment it is summarized in better way as much as possible. Please see in the discussion page number 31 line number 256-263. |
| 14- Discussion, page 26, line 362 to the line 371 needed to be rewritten in summative linked statement | Dear Reviewer, based on your valuable comment it is summarized in better way as much as possible. Please see in the discussion page number 31 line number 264-271. |
| 15- Discussion, page 26, line 372 to the line 375 needed to be rewritten in summative linked statement. | Dear Reviewer, based on your valuable comment it is summarized in better way as much as possible. Please see in the discussion page number 31 line number 272-276. |
| 16- Discussion, page 26, line 377 to the line 379 needed to be rewritten in summative linked statement. | Dear Reviewer, based on your valuable comment it is summarized in better way as much as possible. Please see in the discussion page number 32 line number 277-281. |
| 17- Discussion, page 27, line 388 to the line 394 needed to be rewritten in summative linked statement. | Dear Reviewer, based on your valuable comment it is summarized in better way as much as possible. Please see in the discussion page number 32 line number 282-286 |
| 18- Section for limitation of the study was not seen. | Dear Reviewer, We have outlined the limitation of the study in the revised manuscript. Please see page no 33 & 34 line numbers 317-323 in the revised manuscript. |
| 19- Conclusion, page 29, line 439: this paragraph showed a duplication for the same information in the previous sentences. It is important to summarize and link the information. | Thank you for your valuable feedback. Now we have revised the conclusion based on your feedback. |
| 20- References, page 30, ref. No. 5: it is preferable to show the link of the site. and similar for others that need to state the web link. | Dear reviewer, all the references are corrected to fulfill the journal requirement. |
| **Editor comments** |  |
| The reviewers have raised a number of major concerns. They feel the manuscript should outline the limitations of the study, and they request improvements to the reporting of methodological aspects of the study, for example, regarding the inclusion criteria and more information on the original questionnaire used. | Dear editor, We have outlined the limitation of the study in the revised manuscript. Please see page no 33 & 34 line numbers 317-323 in the revised manuscript.  We have also revised the all methodological aspect of the study including inclusion and exclusion criteria of the study based on the reviewers comments. Please see entire materials and methods and page no 7 line numbers 123-131 in the revised manuscript.  We have also provided the sufficient information about the questionnaire used and observation checklist and source. Please see page no 7 and 8 line numbers 138-162 in the revised manuscript. |
| Journal Requirements:  When submitting your revision, we need you to address these additional requirements. The PLOS ONE style templates can be found at  <https://journals.plos.org/plosone/s/file?id=wjVg/PLOSOne_formatting_sample_main_body.pdf> and  <https://journals.plos.org/plosone/s/file?id=ba62/PLOSOne_formatting_sample_title_authors_affiliations.pdf> | Thank you for your valuable feedback. We appreciate your feedback. We have provided our efforts as much as possible to meet the journal requirements. |
| 1. Please ensure that your manuscript meets PLOS ONE's style requirements, including those for file naming. | Thank you for your valuable feedback. We appreciate your feedback. We have provided our efforts as much as possible to meet the journal requirements. |
| 2. In your Data Availability statement, you have not specified where the minimal data set underlying the results described in your manuscript can be found. PLOS defines a study's minimal data set as the underlying data used to reach the conclusions drawn in the manuscript and any additional data required to replicate the reported study findings in their entirety. All PLOS journals require that the minimal data set be made fully available. For more information about our data policy, please see <http://journals.plos.org/plosone/s/data-availability>.  Upon re-submitting your revised manuscript, please upload your study’s minimal underlying data set as either Supporting Information files or to a stable, public repository and include the relevant URLs, DOIs, or accession numbers within your revised cover letter. For a list of acceptable repositories, please see <http://journals.plos.org/plosone/s/data-availability#loc-recommended-repositories>. Any potentially identifying patient information must be fully anonymized.  Important: If there are ethical or legal restrictions to sharing your data publicly, please explain these restrictions in detail. Please see our guidelines for more information on what we consider unacceptable restrictions to publicly sharing data: <http://journals.plos.org/plosone/s/data-availability#loc-unacceptable-data-access-restrictions>. Note that it is not acceptable for the authors to be the sole named individuals responsible for ensuring data access.  We will update your Data Availability statement to reflect the information you provide in your cover letter. | Thank you for your valuable feedback. We appreciate your feedback. Data available statement is added in the revised manuscript as follows:  **Data Availability**  All the relevant data are presented within the paper. |
| 3. Thank you for stating the following in the Acknowledgments Section of your manuscript:  “We would also like to thank University Grant Commission (UGC) for providing research grant to conduct this study.”  We note that you have provided additional information within the Acknowledgements Section that is not currently declared in your Funding Statement. Please note that funding information should not appear in the Acknowledgments section or other areas of your manuscript. We will only publish funding information present in the Funding Statement section of the online submission form.  Please remove any funding-related text from the manuscript and let us know how you would like to update your Funding Statement. Currently, your Funding Statement reads as follows:  “Manisha B.K. corresponding author received grant with award number MRS-78-79-HS-04 from University Grant Commission (UGC), Nepal. The funder had no role in study design, data collection and analysis, decision to publish, or preparation of the manuscript.  Please include your amended statements within your cover letter; we will change the online submission form on your behalf. | We accept your feedback. The sentences is now removed from the revised manuscript.  Removed statement from Manuscript “We would also like to thank University Grant Commission (UGC) for providing the research grant to conduct this study.” |
| 4. We note you have included a table to which you do not refer in the text of your manuscript. Please ensure that you refer to Table 1 in your text; if accepted, production will need this reference to link the reader to the Table. | Thank you for your valuable comments. Now it is corrected. Please see page no 6 line number 117 in the revised manuscript. |
